# Therapy susceptible germline-related *BRCA 1*-mutation in a case of metastasized mixed adeno-neuroendocrine carcinoma (MANEC) of the small bowel

**DOI:** 10.1186/s12876-018-0803-1

**Published:** 2018-05-31

**Authors:** A. Quaas, D. Waldschmidt, H. Alakus, T. Zander, C. Heydt, T. Goeser, M. Daheim, P. Kasper, P. Plum, C. Bruns, A. Brunn, W. Roth, N. Hartmann, A. Bunck, M. Schmidt, H. Göbel, L. Tharun, R. Buettner, S. Merkelbach-Bruse

**Affiliations:** 1 Institute of Pathology, University of Cologne, Kerpener Strasse 62, 50937 Cologne, Germany; 2 Department of Visceral Surgery, University of Cologne, Kerpener Strasse 62, 50937 Cologne, Germany; 3 Department of Oncology and Hematology, University of Cologne, Kerpener Strasse 62, 50937 Cologne, Germany; 4 Department of Hepato- and Gastroenterology, University of Cologne, Kerpener Strasse 62, 50937 Cologne, Germany; 5 Institute of Neuropathology, University of Cologne, Kerpener Strasse 62, 50937 Cologne, Germany; 6 Institute of Pathology, University of Mainz, Langenbeckstraße 1, 55131 Mainz, Germany; 7 Department of Radiology, University of Cologne, Kerpener Strasse 62, 50937 Cologne, Germany; 8 Department of Nuclear-Medicine, University of Cologne, Kerpener Strasse 62, 50937 Cologne, Germany; 9 Gastrointestinal Cancer Group Cologne, Cologne, Germany

**Keywords:** Small bowel mixed adeno-neuroendocrine carcinoma (MANEC), *BRCA1* mutation, Germline-related, PARP-inhibition, Personalized treatment

## Abstract

**Background:**

Adenocarcinomas or combined adeno-neuroendocrine carcinomas (MANEC) of small bowel usually have a dismal prognosis with limited systemic therapy options. This is the first description of a patient showing a germline-related *BRCA1* mutated MANEC of his ileum. The tumor presented a susceptibility to a combined chemotherapy and the PARP1-inhibitor olaparib.

**Case presentation:**

A 74-year old male patient presented with a metastasized MANEC of his ileum. Due to clinical symptoms his ileum-tumor and the single brain metastasis were removed. We verified the same pathogenic (class 5) *BRCA1* mutation in different tumor locations. There was no known personal history of a previous malignant tumor. Nevertheless we identified his *BRCA1* mutation as germline-related. A systemic treatment was started including Gemcitabine followed by selective internal radiotherapy (SIRT) to treat liver metastases and in the further course Capecitabine but this treatment finally failed after 9 months and all liver metastases showed progression. The treatment failure was the reason to induce an individualized therapeutic approach using combined chemotherapy of carboplatin, paclitaxel and the Poly (ADP-ribose) polymerase- (PARP)-inhibitor olaparib analogous to the treatment protocol of Oza et al.

All liver metastases demonstrated with significant tumor regression after 3 months and could be removed. In his most current follow up from December 2017 (25 months after his primary diagnosis) the patient is in a very good general condition without evidence for further metastases.

**Conclusion:**

We present first evidence of a therapy susceptible germline-related *BRCA*1 mutation in small bowel adeno-neuroendocrine carcinoma (MANEC). Our findings offer a personalized treatment option. The germline background was unexpected in a 74-year old man with no previously known tumor burden. We should be aware of the familiar background in tumors of older patients as well.

## Background

Small bowel carcinomas are rare and account only for 0.5% of all cancers. The 5 year overall survival rates for patients with small-bowel adenocarcinoma is 34.9%. To best of our knowledge no valid prognostic or survival data exist to the mixed adeno-neuroendocrine carcinoma (MANEC)-subtype of the small bowel. There is a strong need for a more effective and personalized systemic treatment option in carcinoma of the small bowel. Most recently Schrock, A et al. [1] described molecular alterations in a very large cohort of 317 small bowel carcinomas. According to this study microsatellite-instability (MSI) was present in 7,6% and are characterized by the following mutations like KRAS (53,6%), APC (26,8%), CDKN2A (14,5%), SMAD4 (17,4%) and BRAF (9,1% but only 10% of these have the typical V600E mutation) as well as ERBB2 mutation in 8,2% of the analysable tumors.

The most important pathway in DNA double strand break (DNA-dbs.) repair is homologous recombination (HR). The HR pathway is dependent on different proteins including *BRCA1* and *BRCA2* and others (e.g. MNR complex, *CtIP, MRE11, RAD51, ATM, H2AX, PALB2, RPA, RAD52*) [2, 3].

Effective DNA repair is essential to maintain DNA integrity – so *BRCA1/2* deficient cells show a high degree of chromosomal instability which increases the risk of malignant transformation [4, 5]. Germline mutations of *BRCA1/2* (gBRCA-mut) were described in carcinoma of the ovary in 5–18% and in some other solid malignant tumors including breast carcinoma, pancreatic carcinoma and less frequent in prostate carcinoma. It became apparent that *BRCA* mutations also occur in sporadic carcinomas and these tumors respond to Poly (ADP-ribose) polymerase- (PARP) inhibition. The full significance of somatic *BRCA* mutations as a biomarker for the prediction of therapeutic response to PARP inhibition is not entirely clear but ovarian cancer patients with a somatic *BRCA* mutation are equally likely to benefit from treatment with PARP inhibitors as those with a germline mutation of *BRCA*. Some authors suggest to perform somatic *BRCA* testing in routine clinical diagnostics of all advanced tumors, which are potentially amenable to PARP inhibitors [3, 6–11].

Each BRCA variant was classified according to the established IARC classification. This classification ranges from class 1 variants (not pathogenic or of no clinical significance) via class 2 (likely not pathogenic or of no clinical significance), class 3 (uncertain), class 4 (likely pathogenic) to class 5 variants (definitively pathogenic) according to different databases (compare: BRCA mutation database: http://arup.utah.edu/database/BRCA/orClinVardatabase:http://www.ncbi.nlm.nih.gov/clinvar/ or Universal mutation database BRCA share: http://www.umd.be/BRCA1/).

### Parallel sequencing

Tumor areas were marked on H&E-stained tissue slides by a pathologist (AQ) and DNA was extracted from corresponding unstained 10 μm thick slides by manual macrodissection. After proteinase K treatment, the DNA was automatically purified using the Maxwell 16 FFPE Tissue LEV DNA Purification Kit (Promega, Madison, USA) on the Maxwell 16 Instrument (Promega) following the manufacturers’ protocol. The DNA concentration was measured using a real-time qPCR-based method.

By parallel sequencing, all tumor manifestations were analyzed for *BRCA1* and *BRCA2* mutation and were further analyzed using a panel of 12 other genes resulting altogether in a total of 452 amplicons. Additionally, in these tumor manifestations microsatellite status was checked using five different mononucleotide markers: BAT25, BAT26, NR-21, NR-22 and NR-27 [12].

Isolated DNA (10 ng each) was amplified with the Human BRCA1 and BRCA2 GeneRead DNAseq Targeted Panel V2 (Qiagen, Hilden, Germany) or with a customized GeneRead DNAseq Alignment and annotation was done using a modified version of a previously described method [13]. Resulting BAM files were visualized using the Integrative Genomics Viewer (IGV; http://www.broadinstitute.org/igv/, Cambridge; USA). A 5% cutoff for variant calls was used and results were only interpreted if the coverage was > 200×.

### Histopathological findings

**Fig. 1:**
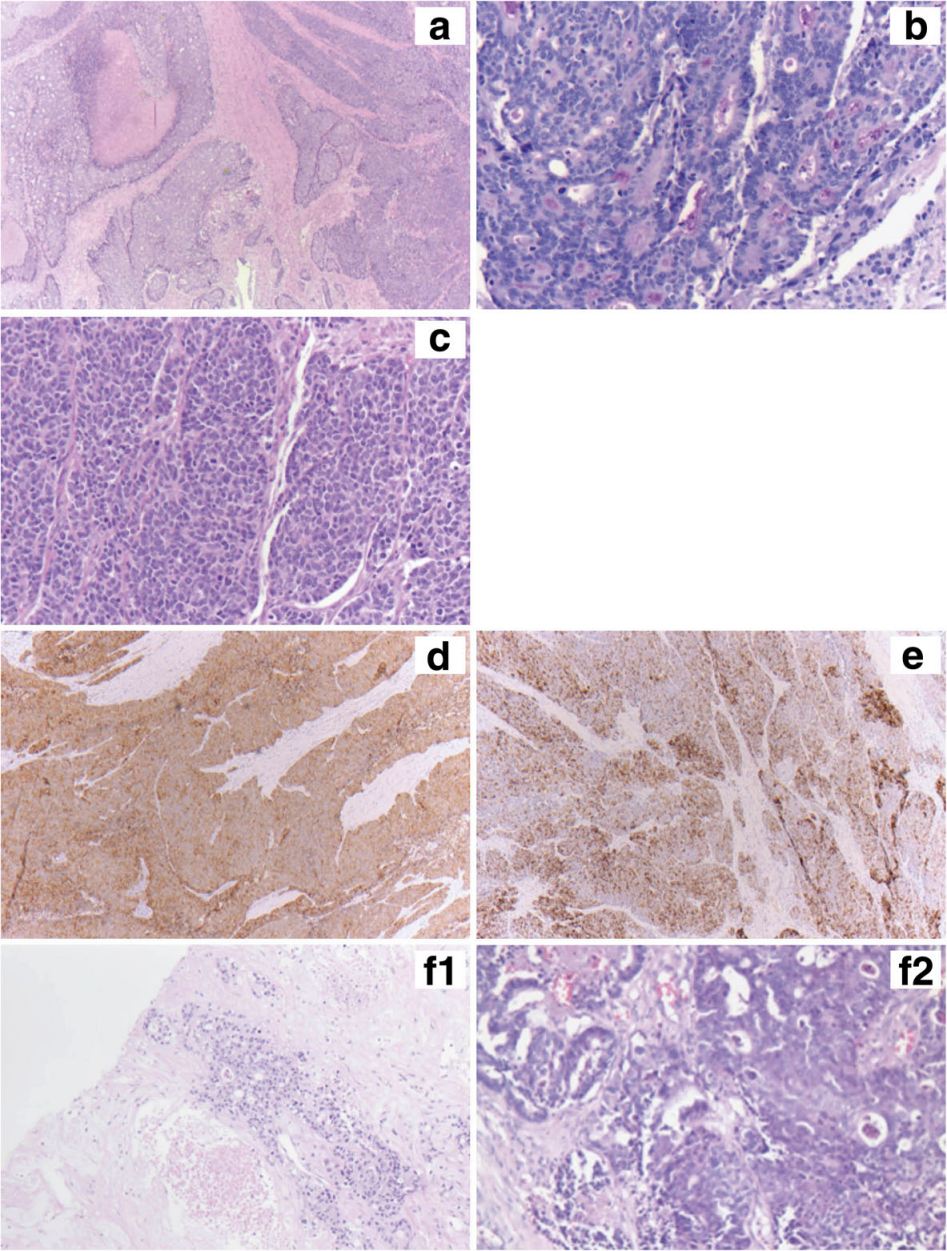
**a** Primary MANEC of the ileum (HE, 2,5×) showing the gland-forming adenocarcinoma-component (left side) as well as the solid forming neuroendocrine carcinoma component (right side); **b** the gland forming adenocarcinoma showing PAS-positive mucins in gland lumina (PAS, 20×); **c** the solid growth-pattern of the neuroendocrine carcinoma component (HE, 20×); **d** Immunohistochemistry using an antibody against synaptophysin – diffuse and strong positivity of all tumor cells in the neuroendocrine-carcinoma component (synaptophysin, 5×); **e** Immunohisto-chemistry using an antibody against chromogranin A – more patchy positivity in the neuroendocrine-carcinoma component (chromogranin A, 5×) **f1** Metastasis in liver and **f2** in the brain, both formed by the adenocarcinoma component (HE, 10× and 20×)

The primary MANEC of the ileum showed two different tumor components: One gland-forming adenocarcinoma-component including PAS-positive mucins in the tumor cell cytoplasm or gland lumina as well as a solid tumor forming neuroendocrine carcinoma-component showing a strong and diffuse immunohistochemical positivity for synatophysin and to a lesser content chromogranin A as well (Fig. 1). The tumor infiltrated the subserosal tissue of the ileum and presented with vessel invasion including a single regional lymph node metastasis. Both, the brain and liver metastasis showed the adenocarcinoma-component.

## Case presentation

We describe the case of a 74-year old man presented to our hospital due to a liver tumor in the segments II and III as well as satellite metastatic lesions in the right liver lobe. After taken a liver biopsy from the tumor the diagnosis of an adenocarcinoma was established and interpreted as an unresectable cholangiocarcinoma, initially lacking the information of the primary tumor in the small intestine. The patient was treated with a mono-chemotherapy of Gemcitabine ((1000 mg/qm d1–8) 12/2015–03/2016) followed by selective internal radiotherapy (SIRT) analogous to the SIRCCA study protocol (ClinicalTrials.gov Identifier: NCT02807181). Because of anemia and poor general condition the administration of cisplatin was withheld. In the further course of treatment an additional tumor site in the ileum was detected and progression with cerebral metastasis occurred. The ileum tumor and one brain metastasis were removed. The ileum tumor was classified as a mixed adeno-neuroendocrine carcinoma (MANEC). Tumor manifestations in the liver and in the brain could be characterized as metastases from the adenocarcinoma component (Fig. 1).

**Fig. 2:**
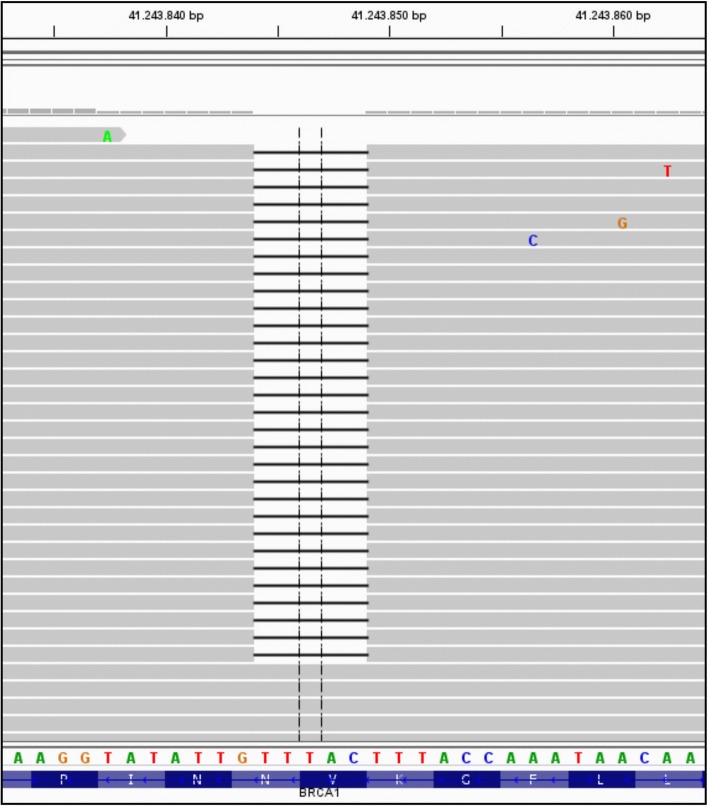
*BRCA* deletion (*BRCA1*: c.3700_3704delGTAAA; p.V1234Qfs*8) as visualized by the IGV (integrative genomics viewer; www.broadinstitute.org/igv/)

In the further course we patient was treated with capecitabine (500 mg 2–0-2, 5d/week) 05–09/2016). This treatment finally failed 4 months later and all liver metastases showed progression. The treatment failure was the reason to analyze the tumor-DNA for molecular alterations using two panels of 14 genes including BRCA 1 and *BRCA 2* (452 amplicons in total; compare versions below). We verified the same *BRCA1* mutation (c.3700_3704delGTAAA p.V1234Qfs*8) with a very high allele frequency up to 96% in all locations using ultra deep sequencing in the formalin-fixed paraffin embedded material (Fig. 2). According to different databases (UMD, ARUP, ClinVar) this *BRCA1* mutation was classified as pathogenic (class 5). The MANEC was microsatellite stable. The mutational hot spots of all other analyzed genes (*KRAS, NRAS, HRAS, BRAF, DDR2, ERBB2, KEAP1, NFE2L2, PIK3CA, PTEN, RHOA* and *BRCA2*) were wild type.

According to the encouraging experiences using combined platin-based chemotherapy and PARP-inhibitors in BRCA-mutated ovarian carcinomas the molecular results have been the trigger for the personalized therapeutic approach using combined chemotherapy of carboplatin, paclitaxel and the PARP1-inhibitor olaparib analogous to the treatment protocol of Oza et al. [14] following a bridging therapy with capecitabine (olaparib 200 mg capsules twice daily, administered orally on days 1–10 of each 21-day cycle) plus paclitaxel (175 mg/m^2^, administered intravenously on day 1) and carboplatin (4 mg/mL per min) in total five cycles for the following 4 months.

**Fig. 3:**
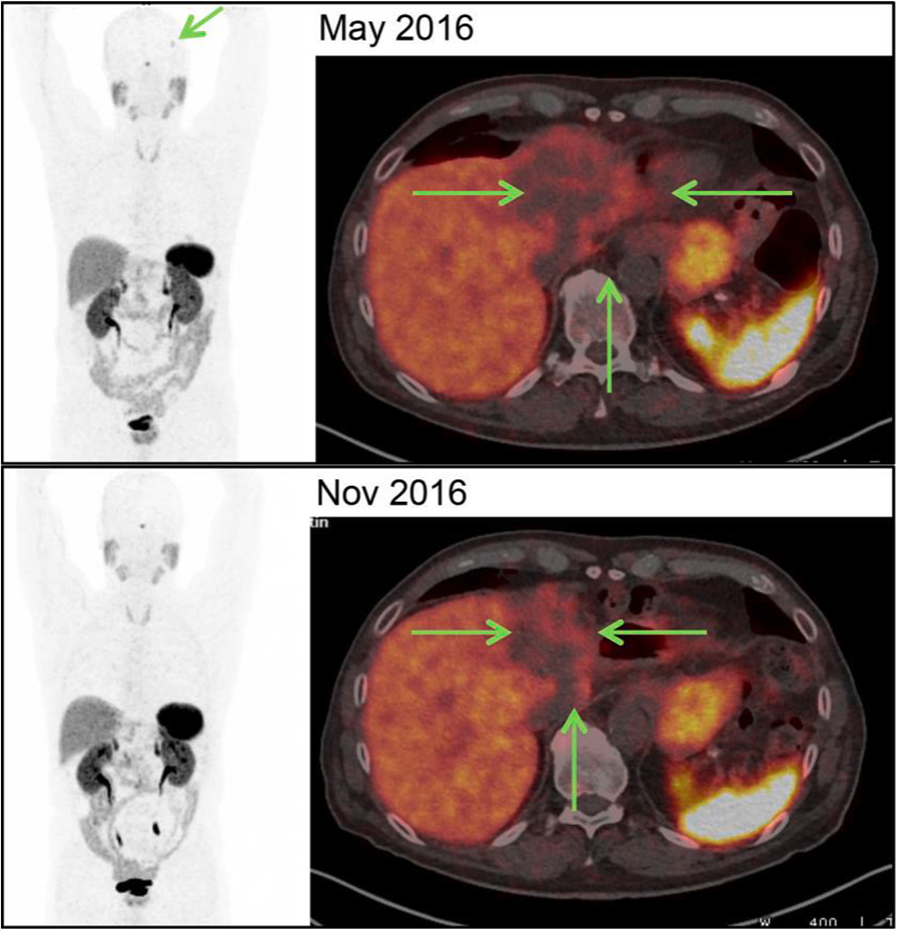
Response to personalized treatment (including olaparib): Ga-68 Dotatate PET/CT. Upper row (May 2016) demonstrating an 8 cm tumor in the left liver lobe below physiologic liver uptake and a SRS-positive brain metastasis. Lower row: (Nov 2016): Follow-up PET/CT with tumor shrinkage to 6 cm and disappearance of the brain metastasis

All tumor manifestations demonstrated with a significant tumor regression in the MINT analysis and Ga-68-Dotatate PET-CT after 3 months of treatment (mind analysis Table 1 and Ga-68-Dotatate PET-CT Fig. 3) – and the persistent highly necrotic liver metastasis of 6 cm could be removed. In his most current follow up from December 2017 (25 months after his primary diagnosis) the patient is in a very good general condition without evidence for further metastases (compare time table, Table 2).

**Table 1:**
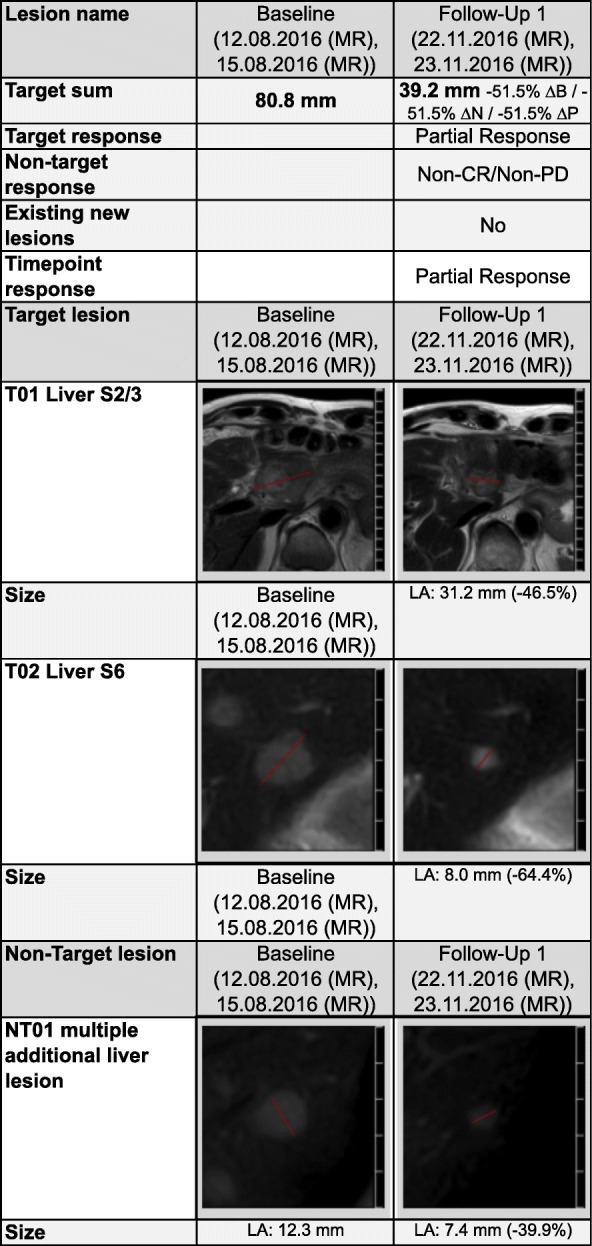
Response to personalized treatment (including olaparib): MINT analysisTumor showed significant regression under personalized treatment approach using olaparib, carboplatin and paclitaxel (data shown before and 3 months after initialization of personalized treatment)

**Table 2:** Time table

Time period	Therapy
12.2015	Pat. presented with liver and brain metastases.
12.2015–03.2016	Gemcitabine mono 1000 mg/qm d1–8 15 and selective internal radiotherapy (SIRT) analogous to the SIRCCA study protocol.Excision of the tumor in the ileum and one brain metastasis (02.2016)
05.2016–09.2016	Capecitabine 500 mg (2–0-2, 5d/week)
12.09.16	1. Cycle: Olaparib (200 mg capsules twice daily, administered orally on days 1–10 of each 21-day cycle) plus paclitaxel (175 mg/m^2^, administered intravenously on day 1) and carboplatin (4 mg/mL per min)
04.10.2016	2. Cycle: Olaparib (200 mg capsules twice daily, administered orally on days 1–10 of each 21-day cycle) plus paclitaxel (175 mg/m^2^, administered intravenously on day 1) and carboplatin (4 mg/mL per min)
31.10.2016	3. Cycle: Olaparib (100 mg capsules twice daily (reduced dosage), administered orally on days 1–10 of each 21-day cycle) plus paclitaxel (175 mg/m^2^, administered intravenously on day 1) and carboplatin (4 mg/mL per min)
05.12.2016	4. Cycle: Olaparib (100 mg capsules twice daily (reduced dosage), administered orally on days 1–10 of each 21-day cycle) plus paclitaxel (175 mg/m^2^, administered intravenously on day 1) and carboplatin (4 mg/mL per min)
09.01.2017	5. Cycle: Olaparib (100 mg capsules twice daily (reduced dosage), administered orally on days 1–10 of each 21-day cycle) plus paclitaxel (175 mg/m^2^, administered intravenously on day 1) and carboplatin (4 mg/mL per min)
02.2017	liver metastases could be removed
12.2017	Last CT-scan staging: still no further tumor manifestations/still no metastasis

## Discussion and conclusion

To the best of our knowledge this is the first description of pathogenic and treatable germline-related *BRCA1* mutation in mixed adeno-neuroendocrine carcinoma (MANEC) of the small bowel. There was no known personal history of a previous malignant tumor. Nevertheless, the high allele frequency of the *BRCA1* mutation in all tumor manifestations suggested a germline background. Consequently, we tested histologically tumor-free ileum mucosa and were able to detect the same *BRCA1* mutation with an allele frequency of 48%. This finding clearly identifies the mutation as germline and a deletion of the wild type *BRCA1* allele in the tumor. The homogeneous distribution of *BRCA1* mutated tumor cells in the primary tumor and in the different metastasis analyzed here suggests a clonal origin and a causative role of this second hit. Even though a g*BRCA1* mutation could be a marker for prostate cancer we did not find any indications for prostate cancer [7].

According to treatment protocols established in BRCA mutated ovarian carcinomas we successfully applied combined chemotherapy of carboplatin, paclitaxel and the PARP1-inhibitor olaparib. Initially, we assumed a somatic background of the *BRCA1* mutation in our treated patient, because there was no known personal history of a prior malignant tumor in the 74 year-old man. The germline background of the *BRCA1* mutated small bowel carcinoma (like in ovarian adenocarcinoma) is especially promising for a successful combination therapy using platinum-based chemotherapy and a PARP inhibitor (e.g. Olaparib). It has to be questioned whether the somatic pathogenic *BRCA* mutation itself is the main predictor of an effective PARP inhibition [3].

Beyond *BRCA1/2* somatic mutations or the promoter methylation of *BRCA* some other alterations are potential markers of an effective PARP inhibition (like microsatellite-instable cancers with MRE11-dominant negative mutations, *PTEN* deficiency, *ATM* mutation and *FANCF* promoter methylation) [15].

Additionally, first evidence came up that *gBRCA* mutated ovarian carcinoma are sensitive to therapies using immune-checkpoint inhibition most likely due to its higher mutational load compared to wild type ovarian carcinoma [16]. It remains to be shown whether our *BRCA* mutated MANEC also benefit from immune-checkpoint inhibition as an additional future treatment option.

Our findings are remarkable in many ways: Here we provide first evidence of a therapy susceptible *BRCA*1 mutation in a metastasized mixed adeno-neuroendocrine carcinoma (MANEC) of small bowel.

The germline background of our patient was unexpected because he is a 74-year old man with no previously known tumor burden. Currently, there is increasing evidence that the first germline associated tumor manifestation (e.g. endometrial carcinoma in Lynch syndrome) may occur beyond the sixth decade of living which means that we should be aware of the familiar background in tumors of older patients as well [17].

## Abbreviations

BRCA1: Breast cancer gene 1
MANEC: Mixed adeno-neuroendocrine carcinoma
PARP: Poly (ADP-ribose) polymerase
